# A Gap Analysis Methodology for Collecting Crop Genepools: A Case Study with *Phaseolus* Beans

**DOI:** 10.1371/journal.pone.0013497

**Published:** 2010-10-20

**Authors:** Julián Ramírez-Villegas, Colin Khoury, Andy Jarvis, Daniel Gabriel Debouck, Luigi Guarino

**Affiliations:** 1 Decision and Policy Analysis Program, International Center for Tropical Agriculture (CIAT), Cali, Colombia; 2 Global Crop Diversity Trust, Rome, Italy; 3 Adaptation to Progressive Climate Change, Challenge Program on Climate Change, Agriculture and Food Security (CCAFS), Copenhagen, Denmark; 4 Bioversity International, Regional Office for the Americas, Cali, Colombia; 5 Genetic Resources Unit, International Center for Tropical Agriculture (CIAT), Cali, Colombia; University College London, United Kingdom

## Abstract

**Background:**

The wild relatives of crops represent a major source of valuable traits for crop improvement. These resources are threatened by habitat destruction, land use changes, and other factors, requiring their urgent collection and long-term availability for research and breeding from *ex situ* collections. We propose a method to identify gaps in *ex situ* collections (i.e. gap analysis) of crop wild relatives as a means to guide efficient and effective collecting activities.

**Methodology/Principal Findings:**

The methodology prioritizes among taxa based on a combination of sampling, geographic, and environmental gaps. We apply the gap analysis methodology to wild taxa of the *Phaseolus* genepool. Of 85 taxa, 48 (56.5%) are assigned high priority for collecting due to lack of, or under-representation, in genebanks, 17 taxa are given medium priority for collecting, 15 low priority, and 5 species are assessed as adequately represented in *ex situ* collections. Gap “hotspots”, representing priority target areas for collecting, are concentrated in central Mexico, although the narrow endemic nature of a suite of priority species adds a number of specific additional regions to spatial collecting priorities.

**Conclusions/Significance:**

Results of the gap analysis method mostly align very well with expert opinion of gaps in *ex situ* collections, with only a few exceptions. A more detailed prioritization of taxa and geographic areas for collection can be achieved by including in the analysis predictive threat factors, such as climate change or habitat destruction, or by adding additional prioritization filters, such as the degree of relatedness to cultivated species (i.e. ease of use in crop breeding). Furthermore, results for multiple crop genepools may be overlaid, which would allow a global analysis of gaps in *ex situ* collections of the world's plant genetic resources.

## Introduction

Crop wild relatives (CWR) are wild plant species sharing relatively recent common ancestry with cultivated plants. CWR typically possess wide diversity, much of it not found in the crop, and this diversity may be introgressed into the crop by plant breeders, with the ease of transfer of genes generally dependent on the degree of relatedness between the wild species and the domesticate [1], [2]. Wild relatives have provided to crops traits such as pest and disease resistance, tolerance to abiotic stresses, increased yield, male sterility, and quality, increasing the value and sustainability of banana, barley, beans, cassava, chickpea, lettuce, maize, oats, pearl millet, potatoes, rice, sugar cane, sunflower, tomato, and wheat production, among others. In the past 20 years, there has been a steady increase in the rate of release of cultivars containing genes from CWR, and their contribution should only increase as the development of molecular technologies makes identification and utilization of diverse germplasm more efficient [2]–[5].

Plant breeders obtain CWR material from genebanks. However, major gaps in the genetic diversity of important crop genepools remain to be filled in *ex situ* germplasm collections. These gaps are particularly evident for non-cereal crops (e.g. legumes, roots and tubers, vegetables), and for wild and weedy forms [6]–[8]. Maxted and Kell [7] estimated that 94% of European CWR species are completely missing from *ex situ* collections. At the same time, habitat destruction, invasive species, urbanization, and the shift from traditional to industrial agricultural practices, among other factors, continue to threaten PGR, and climate change is projected to impose further pressures on both wild and agricultural ecosystems [9]–[15].

Clearly, much collecting of CWR diversity is still required. Unfortunately international efforts in collecting plant genetic resources in general have been in decline in recent decades [16]. The recent coming into force of the International Treaty on Plant Genetic Resources for Food and Agriculture is, however, expected to provide impetus for the development of an integrated, effective, efficient, global approach to conserving PGR. The development of strategic planning approaches will be necessary to prioritize PGR for collecting as part of such a rational global system.

Gap analysis refers to a systematic method of analyzing the degree of conservation of taxa, in order to identify those locations, taxa, and particular traits (adaptations) un- or under- secured in conservation systems [17]. Nabhan [18] identified four ways by which gap analysis techniques may lead to better collecting and conservation: targeting localities where sets of species absent from existing collections can be obtained with least effort and cost; determining which areas are ‘under-collected’ or ‘over-collected’ for germplasm relative to the known distribution of a taxon; locating which regions have the greatest or most dissimilar species richness compared with other regions; and outlining the ecological amplitudes of each species so that a wider representation of the ecotypes or genetically adapted populations of each can be sampled.

Geographic Information Systems (GIS) technologies have enabled a better understanding of species distributions and of the representativeness of germplasm collections, and have contributed to conservation planning of wild species, CWR, and domesticates [17]–[28]. Pioneering the use of these tools in conservation, Jones et al. [25] successfully predicted the location of populations of wild common bean (*Phaseolus vulgaris*), based on climatic suitability. Significant developments have occurred in recent years in the application of GIS to PGR conservation planning, including the development and validation of various approaches to niche modeling, new analysis tools and extensions, and better access to geographic information, results and approaches [29].

We propose here a gap analysis method designed to inform planning of germplasm collecting for *ex situ* conservation, based upon available information resources, using GIS. The distributions of *ex situ* collections are compared to GIS-modeled taxon distributions based on both herbarium and genebank data. The gross total number of germplasm accessions, as well as the distribution (geographical and environmental) of those accessions, are compared against modeled distributions in order to identify gaps in *ex situ* conservation coverage. These results form the basis for a prioritization of taxa across the genepool for collecting, and the identification of the highest priority locations (i.e. diverse and under-represented areas) for the most efficient and effective collecting, in order to further enhance *ex situ* holdings. Our model genepool is *Phaseolus*.

The genus *Phaseolus* originated in the tropics and subtropics of the New World, and contains up to 81 species and 34 infraspecific taxa [2], [30]–[32], having undergone a series of revisions, notably in association with members of *Vigna*, which have included splitting some species into new genera (e.g. *Strophostyles*, *Dysolobium*, *Macroptilium*, *Minkelersia* and *Alepidocalyx*) [27]. The main centers of diversity for the genus are in wide Mesoamerica (from southern USA, Mexico, and Central America down to Panama), the northern Andean region (Colombia to northern Peru), and the central Andes (northern Peru, Bolivia to northwest Argentina). Of these, the Mesoamerican centre is the richest in species [30], [32]–[34].

*Phaseolus* has five domesticated species, each a result of an independent domestication process: *P. vulgaris* L.- common bean; *P. lunatus* L.- lima bean; *P. coccineus* L.- runner bean; *P. acutifolius* A. Gray - tepary bean; and *P. dumosus* Macfady - year bean. The genus has been cultivated for over 7000 years, and each of the cultivated species has distinct ecological adaptations [35]. Common bean is the world's most important legume for food production and security, and represents 50% of the grain legumes consumed worldwide, reaching primary importance in the staple diet of over 500 million people, especially for its protein content [31], [36]. Common bean is now grown on over 27 million hectares globally, producing over 20 million tons [37].

Diversity in *Phaseolus* in relation to the cultivated species is organized into genepools based on phylogenetic relationships [38], [39]. The primary genepool of cultivated species includes both cultivars and wild populations, hybrids of which are generally fully fertile with no major reproductive barriers. *P. vulgaris* also allows a measure of interspecific hybridization with species in its secondary genepool. *P. lunatus* and *P. acutifolius* appear less capable of gene exchange with related species [40].

Like many important food crops, cultivars of common bean have a narrow genetic base, attributable to the genetic bottleneck accompanying the domestication process, stringent quality requirements in the market, limited past use of exotic germplasm in breeding, and conservative breeding programs for the crop [2]. Interspecific and wide intraspecific crossing have been useful strategies for crop improvement, but given the still limited genetic base, more along these lines is needed. Useful alleles for many agronomic traits deficient in common bean cultivars, including resistance to storage insects, leafhoppers, ascochyta blight, common bacterial blight, white mold, bean common mosaic virus, rust, drought, and soil fertility problems, as well as early maturity, adaptation to higher latitudes, upright plant type, pod quality, and seed yield have been identified in wild common bean and species in the secondary and tertiary genepools, and utilized in breeding programs [2], [41]–[43]. Wild common bean has also contributed high protein digestibility [44] and nodulation [45] traits. Despite the increasing utilization of CWR in common bean breeding, Singh [2] estimated as much as 90% of the genetic variability available in the primary genepool and related species as under- or not utilized. Widening of genetic diversity in the other *Phaseolus* crop species may also prove important. The domestication of tepary bean involved a severe genetic bottleneck event, leading to a particularly low level of genetic diversity in the crop [46]–[48].

Close to 250 *ex situ* germplasm collections of *Phaseolus*, holding approximately 260,000 accessions, have been established worldwide [16]. The vast majority of these accessions are of common bean, with much smaller collections of the other cultivated species, and a small percentage of wild species. The largest collections of CWR of *Phaseolus* are held in the international collection managed by the Consultative Group on International Agricultural Research (CGIAR), with close to 2000 accessions [49], and in the United States National Genetic Resources Program, with close to 500 accessions) [50].

## Methods

An eight-step gap analysis process is presented, which attempts to evaluate conservation deficiencies at three different levels: (1) taxonomic, (2) geographic and (3) environmental. The aim is to define the extent to which current genebank holdings represent total genetic diversity within a genepool. We apply the protocol to all the wild members of the genus *Phaseolus*.

Based upon the average of overall taxonomic, geographic, and environmental coverage factors, the method produces a table outlining the high, medium and low priority species for collecting. From this table, potential collecting areas for high priority species may be highlighted, and overlapping high priority regions for the collection of multiple taxa identified. In detail, the method is as follows:

### 1. Determination of target taxa, delineation of target area and harvesting of occurrence data:

This involves five steps:

- Identification of the target cultivated species.
- Taxonomical review of all CWR related to the cultivated species, and analysis of relatedness to the domesticated species using the concept established by Maxted *et al.* [51].
- Creation of a database containing as many records as possible both of genebank accessions and herbarium specimens, along with (when available) their respective passport data, specifically the names of the places of collection and coordinates (i.e. latitude and longitude). Samples listed as weedy or cultivated are not included in the database.
- Cross-check, verification, and correction of geographic references (coordinates) through thorough review of data and use of verification tools such as BioGeomancer (www.biogeomancer.org) [52], Google Earth, and high detail physical maps of localities, and strict selection only of verified geo-referenced samples for distribution modeling, as the quality of location data strongly affects the performance of niche modeling techniques [53].
- Determine target area for the gap analysis: based upon the native (wild) distribution of the target taxa. Depending on the genepool, the area can range from a small region within a country to the entire world.

### 2. Determination of sampling deficiencies at the taxon level

A gross representativeness of genebank accessions for each taxon is calculated using the ‘sampling representativeness score’ (SRS, Eqn. 1), comparing total germplasm accessions to herbarium records. 
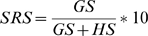
(Eqn. 1)


SRS is calculated as the number of germplasm samples (GS) divided by the total number of samples, i.e. the sum of germplasm plus herbarium samples (HS), regardless of whether samples contain location data. SRS therefore permits a general estimation of adequacy of germplasm holdings of each taxon based upon all available data. In the case that a taxon has no genebank samples, it is listed as a “high priority species” for collecting by setting the FPS (see step 7 below) to 0.

In the rare case that for a particular taxon there is obviously deficient herbarium sample data in comparison to germplasm records, the analysis should eliminate SRS as an input for that taxon, as its inclusion would overestimate adequacy of conservation. Mapping of herbarium samples and genebank accessions can be performed (e.g. using DIVA-GIS (version 7.1.70) [29], [54]) in order to provide a general geographic assessment of the available data.

### 3. Create potential distribution models for taxa

Potential distributions of taxa are calculated using the maximum entropy (Maxent) model [55], with a set of bioclimatic variables and species presence data as inputs. We do not consider the total number of samples with coordinates but the number of different populations represented by those samples (unique locations) [55]–[58]. We use Maxent due to its precision and confidence when predicting species distributions [55]–[62]. Default features are used in Maxent, in which complexity of the models (represented by the number of terms and the type of interactions between environmental variables) depend upon the number of input data points [55], [59]. Background points for model training equal 10,000 random points over the distributional range of the genepool in order to avoid overfitting [63], [64].

As the Maxent distribution is generally broader than the real distribution of the species, the modeled distribution is further refined by selecting only known native areas and high probability zones, which generally are defined as the most climatically suitable for the taxon, thus avoiding over-estimation of the realized niche [63], [65]. The potential distribution is limited to the native area reported in the literature and then thresholded using the ROC (receiver operating characteristic) curve plot-based approach (point on the ROC curve [sensitivity vs. 1-specificity] which has the shortest distance to the top-left corner [0,1] in the ROC plot) [55], [59], [66]. We use this threshold as it provides a decent omission rate, is taxon-specific and shows better performance than other thresholds when predicting potential presence [66]. We call this thresholded modeled distribution the “potential distribution coverage”.

Based on the above, for each taxon, we report three model performance metrics: (1) the 25-fold average area under the ROC curve (AUC) [55], [61], [64], [66] of test data (ATAUC), (2) the standard deviation of the test AUC of the 25 different folds (STAUC), and (3) the proportion of the potential distribution coverage with standard deviation above 0.15 (ASD15). Maxent models with ATAUC above 0.7 [66], STAUC below 0.15, and ASD15 below 10% can be considered “accurate and stable” and are thus used in further calculations. We use three measures of model accuracy as the use of AUC alone might mislead the interpretation given the sensitivity of this measure to spatial autocorrelation [67], [68].

For those taxa for which the Maxent model training fails or is inaccurate or unstable, we assign a priority to the taxa using the following criteria:

- As with step (2), taxa with no genebank samples are listed as “high priority species” for collecting by setting the FPS (see step 5 below) to 0.
- Taxa with genebank samples but no herbarium samples with verified location data are listed as “high priority species” for collecting, as more data are needed in order to perform the analysis. Taxa with such paucity of herbarium records are likely to also have limited germplasm conserved, and are therefore very likely to be “high priority species”. However, these taxa might differ from taxa in (3a) since they already have at least one genebank accession, which certainly permits some type of analyses (e.g. genetic diversity). These taxa are thus differentiated from taxa in (3a) by a flag in the final priorities table (see results).
- Taxa with genebank samples and one or more herbarium samples with verified location data are assessed using the area of the convex hull around all known populations (unique locations) of the taxon in lieu of potential distribution coverage. We use the convex hull since, particularly for taxa with very limited occurrence data; it provides a polygon resembling the type of area produced by the Maxent distribution model.

At this point, the potential distribution coverage for all taxa (for which a niche model is possible) may be mapped together in order to display the distribution of the genus, and a richness map along with an uncertainty map (i.e. maximum standard deviation of probabilities among the species that are present in each pixel) for the genepool may be calculated from the results.

### 4. Geographic coverage assessment

The adequacy of geographic coverage of genebank accessions is calculated as a ‘geographic representativeness score’ (GRS, Eqn. 2), assessed by comparing the taxon potential distribution coverage with the genebank samples geographic coverage, modeled using the ‘circular area statistic with a 50 km radius’ (CA50) value [29]. 
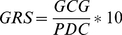
(Eqn. 2)


GRS is thus the geographic coverage of germplasm collections (*GCG*) divided by the potential distribution coverage of the taxon under analysis (*PDC*). The higher the GRS, the higher the representativeness of genebank collections in relation to the potential distribution of the taxon.

### 5. Determination of environmental gaps

The adequacy of environmental coverage of genebank accessions is calculated as an ‘environmental representativeness score’ (ERS, Eqn. 3), assessed by comparing the germplasm samples in relation to the full environmental range of the modeled taxon distribution. The same set of climatic layers used for developing the potential distribution coverage are standardized to have an average of zero and a standard deviation of 1 in order to perform a principal components analysis. The first two of these spatially explicit components (which normally account for more than 70% of the spatial variability) are reclassified into twenty equal classes.
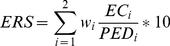
(Eqn. 3)


For these two principal components (i = 2), ERS is calculated as the environmental coverage (i.e. number of different classes) of germplasm collections (EC) divided by the potential environmental coverage of the taxon under analysis (PED), times the weight (w) of the principal component (weights of the two components are re-scaled so that the sum of their weights is 1). If the total variation explained by the first two components is too small (i.e. less than 70%), additional components can be included in the analysis, and should be weighted accordingly.

### 6. Rarity of each species based on environmental variables determination

All records for the genepool (i.e. GS + HS for all taxa combined) are plotted against a specific environmental variable or linear combination of variables (i.e. vector or principal component) to identify taxa with records falling in rare environmental classes (i.e. extremes of the distribution). We assume that the frequency of the data presents a normal distribution and ‘environmentally rare’ taxa are those located in sites where extreme environmental conditions are found (tails of the distribution - 5^th^ [NS_P5_] and 95^th^ [NS_P95_] percentiles). A numeric value (environmentally rare taxa score, ERTS, Eqn. 4) is calculated for each taxon as the number of populations in rare environments divided by the total number of populations of that taxon. 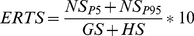
(Eqn. 4)


As this step of the gap analysis should be conducted only when there is sufficient data for all the taxa under analysis in order to avoid bias in the results (an abundant number of populations so that a histogram can be calculated), usually it will not be included in the overall assessment. We suggest that this step can be usefully included for the assessment of a specific subset of well-sampled species.

### 7. Numeric assessment to determine the priority of collecting for *ex situ* conservation for each taxa

All level-specific representativeness scores (SRS, GRS, ERS, and if possible ERTS) are averaged with equal weight to obtain a final score of prioritization of species. The ‘final priority score’ (FPS), is then used to classify taxa according to the following ranges: (1) as high priority species if the FPS is between 0 and 3, (2) as medium priority species if the FPS is between 3.01 and 5, (3) as low priority species if the FPS is between 5.01 and 7.5, and (4) as well conserved species (no need for further collection) if the FPS is between 7.51 and 10. All taxa flagged as high priority in steps (2) and (3) are included in the list of high priority taxa to be further collected.

### 8. Prioritization of geographic areas for collecting germplasm

The potential collection zones for each high priority species are identified separately and then combined to highlight those zones where gaps for multiple species overlap (“collection gap richness”). This is done through the following steps:

- Identify un-collected zones for each taxon by comparing the potential distribution coverage with the current geographic coverage of germplasm collections (CA50). Areas where the taxon is potentially present but already sampled are dismissed at this stage; the remaining areas are highlighted as uncollected.
- Four products treating all mappable high priority taxa are finally produced: (1) individual maps showing potential collecting zones of all high priority taxa, (2) a map of collection gap richness: the number of different taxa that can be collected in each 2.5 arc-minutes (∼5 km at the Equator) grid cell, (3) a map showing the maximum standard deviation of high priority taxa (derived from the 25-fold Maxent model training procedure) in each pixel, and (4) a map of the maximum distance of each pixel to the nearest accession (this calculation is done taxon-by-taxon and then aggregated into a single map output, by calculating the maximum of all ‘high priority taxa’.

### Testing the gap analysis methodology

The methodology relies on available data and utilizes modeling tools, and is therefore vulnerable to the quantity and quality of input data and the limitations of the modeling applied. In order to test the quality of the results, we have compared them to expert opinion, as following:

1. Identify one or more experts on the target taxa (i.e. genepool)
2. Query the selected expert(s) to provide
  1. A ranking of taxa for importance for conservation: To achieve this, the list of taxon names under analysis is sent to the expert(s), who is asked to provide a rating from 1 to 10 for each taxon (where 1 corresponds to a very high priority [i.e. an incomplete collection], and 10 corresponds to the lowest priority [i.e. a complete collection]), without having seen the results of the gap analysis. The expert is requested to rate taxa strictly on the basis of adequacy of *ex situ* holdings for the taxon.
  2. The expert is then shown the results of the analysis and is asked to give general comments on the validity of the taxa and geographic prioritizations.
3. Compare the expert and method-based prioritization of each taxon using the relative difference (RD) between the expert priority score (EPS) and the gap analysis FPS, with respect to the total maximum possible difference [Eqn. 5]

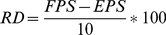
(Eqn. 5)RD is calculated for each taxon and the number of taxa with very similar ratings (−30<RD<30%), the number of taxa somewhat similar ratings (−50%<RD<50%), and the number of taxa with very different ratings (RD <−70% and RD >70%) are then counted. We also plot the FPS and the EPS in a scattergram and calculate both the Spearman correlation coefficient and the P-value of the Spearman correlation coefficient. With these metrics, we aim to provide a general evaluation of the gap analysis method in identifying high priority taxa in comparison to best available expert knowledge.

## Results

### 1. Determination of target taxa, delineation of the target area and harvesting of occurrence data

We conducted a literature review for the *Phaseolus* genus [30], [32], [69]–[71], checked against genepool experts (Debouck) and created a complete list of taxonomically verified species. We used the concept established by Maxted et al. [51], including with equal weight all taxa belonging to taxon groups 1 to 4 of the genepool.

According to a recent revision of the *Phaseolus* genepool [32], there are 81 species and 34 infra-specific taxa, totaling 115 taxa within the genepool. With various species synonyms and historical revisions [27], [30], [32], specimen identification and data availability issues persist. Although taxonomically verified herbarium specimens provided the bulk of the data used in the analysis, we also rely on the specimen identification performed by the individual holding institutions. Based on the recent history of *Phaseolus* taxonomy, we made the following changes to the determination of specimens used in the data: Any variant within *P. polymorphus* Wats. was considered as *P. polymorphus*, and the same was done for *P. coccineus* L. and *P. leptostachyus* Benth. [34]. The variants *P. polystachyus* subsp. *smilacifolius* (Pollard) Freytag and *P. polystachyus* subsp. *sinuatus* (Nuttall ex Torrey & Gray) Freytag were considered as separated species (*P. smilacifolius* and *P. sinuatus,* respectively), and the species *P. pyramidalis* Freytag and *P. palmeri* Piper were merged into *P. grayanus* Woot. & Standl. The only infraspecific taxa that were considered were those of wild teparies (*P. acutifolius*) and those of *P. maculatus*, for which there was not enough evidence for merging into single species. For taxa with ongoing taxonomic uncertainty (e.g. *P. neglectus* Hermann), we followed Debouck [32] and CIAT's Genetic Resources Unit genebank practice. After these modifications, a total of 85 taxa were finally listed, including 81 species and 4 infraspecific taxa.

We gathered data from all known available sources, including primary datasets accessed directly from herbaria and genebanks, as well as online global databases, such as the Global Biodiversity Information Facility (GBIF, www.gbif.org), the System-wide Information Network for Genetic Resources (SINGER, www.singer.cgiar.org) database held by the CGIAR, and the United States Department of Agriculture (USDA) Germplasm Resources Information Network (GRIN, www.ars-grin.gov) database (Table 1).

**Table 1:** List of institutions from which data was harvested.

Institution	Number of records with coordinates	Number of records without coordinates
***Genebank accessions***		
Bioversity International	7	51
CIAT-Genetic Resources Unit (via SINGER)	2278	250
German National Resource Centre for Biological Material (DSMZ)	0	2
International Livestock Research Institute (ILRI)	0	271
Leibniz Institute of Plant Genetics and Crop Plant Research (IPK)	0	21
National Vegetable Germplasm Bank, Mexico (BANGEV)	7	0
Native Seeds/SEARCH (NSS)	37	1
Plant Breeding and Acclimatization Institute (IHAR)	0	17
US National Plant Germplasm System (NPGS-GRIN)	1081	771
**Sub-total**	3410	1384
***Herbarium samples***		
A Database System for Systematics and Taxonomy (SysTax)	2	49
Arizona State University Vascular Plant Herbarium	829	172
Bernice Pauahi Bishop Museum	0	1
Botanic Garden and Botanical Museum Berlin-Dahlem	0	1
Cahiers de Phaseologie (DGD)	1486	182
Canadian Biodiversity Information Facility	0	1
Colorado State University Herbarium (CSU)	33	4
Comision nacional para el conocimiento y uso de la biodiversidad (CONABIO)	1049	360
Dutch national node of the Global Biodiversity Information Facility (NLBIF)	0	25
Fairchild Tropical Botanic Garden Virtual Herbarium	2	18
GBIF-Spain	0	5
GBIF-Sweden	0	6
Harvard University Herbaria	2	86
Herbarium of the University of Aarhus	8	0
Instituto de Biologia, Universidad Nacional de Mexico, (IBUNAM)	0	2
Instituto de Ciencias Naturales	22	68
Instituto Nacional de Biodiversidad (Costa Rica)	78	0
Integrated Taxonomic Information System (ITIS)	8	0
Louisiana State University Herbarium	0	9
Missouri Botanical Garden	713	621
Museo Nacional de Costa Rica	100	45
Muséum national d'histoire naturelle et Réseau des Herbiers de France	4	0
National Botanic Garden of Belgium (NBGB)	70	20
National Museum of Natural History	28	64
NatureServe	0	134
NavNat, GE, FR	2	0
New Mexico Biodiversity Collections Consortium	0	112
New York Botanical Garden (NYBG)	7	4
Royal Botanic Gardens, Kew	1	2
The Deaver Herbarium, Northern Arizona University	8	0
University of Alabama Biodiversity and Systematics	6	0
University of California, Davis	0	7
University of Connecticut	1	0
University of Kansas Biodiversity Research Center	1	3
USDA Plants	402	65
Utah Valley State College (UVSC)	1	3
**Sub-total**	4863	2069
**Total (genebank accessions and herbarium samples)**	8273	3453

Data were available for all taxa, including the 81 species, 2 subspecies and 2 varieties. The entire dataset was carefully geographically verified and corrected using BioGeomancer, and, when possible, new geographic references (coordinates) were added to the passport data. The final dataset contained 11,442 records, of which 6,926 (60.5%) had coordinates or enough location data to obtain coordinates, and 4,516 (39.5%) samples had no location data or coordinates.

The analysis was based on the native range for the genus throughout the Americas (northeastern United States to northern Argentina, including the Caribbean and the Galapagos Islands) [30], [50]. Records outside the boundaries of the Americas, as well as those listed as weedy or cultivated, were deleted and a final dataset was produced for analysis. The average total number of samples per taxon was 144.8, but data was unevenly distributed. Samples were predominantly concentrated in wild progenitors of domesticated species (i.e. *P. acutifolius*, *P. coccineus*, *P. dumosus*, *P. lunatus*, *P. vulgaris*), comprising about 55% of the total records.

**Figure 1:**
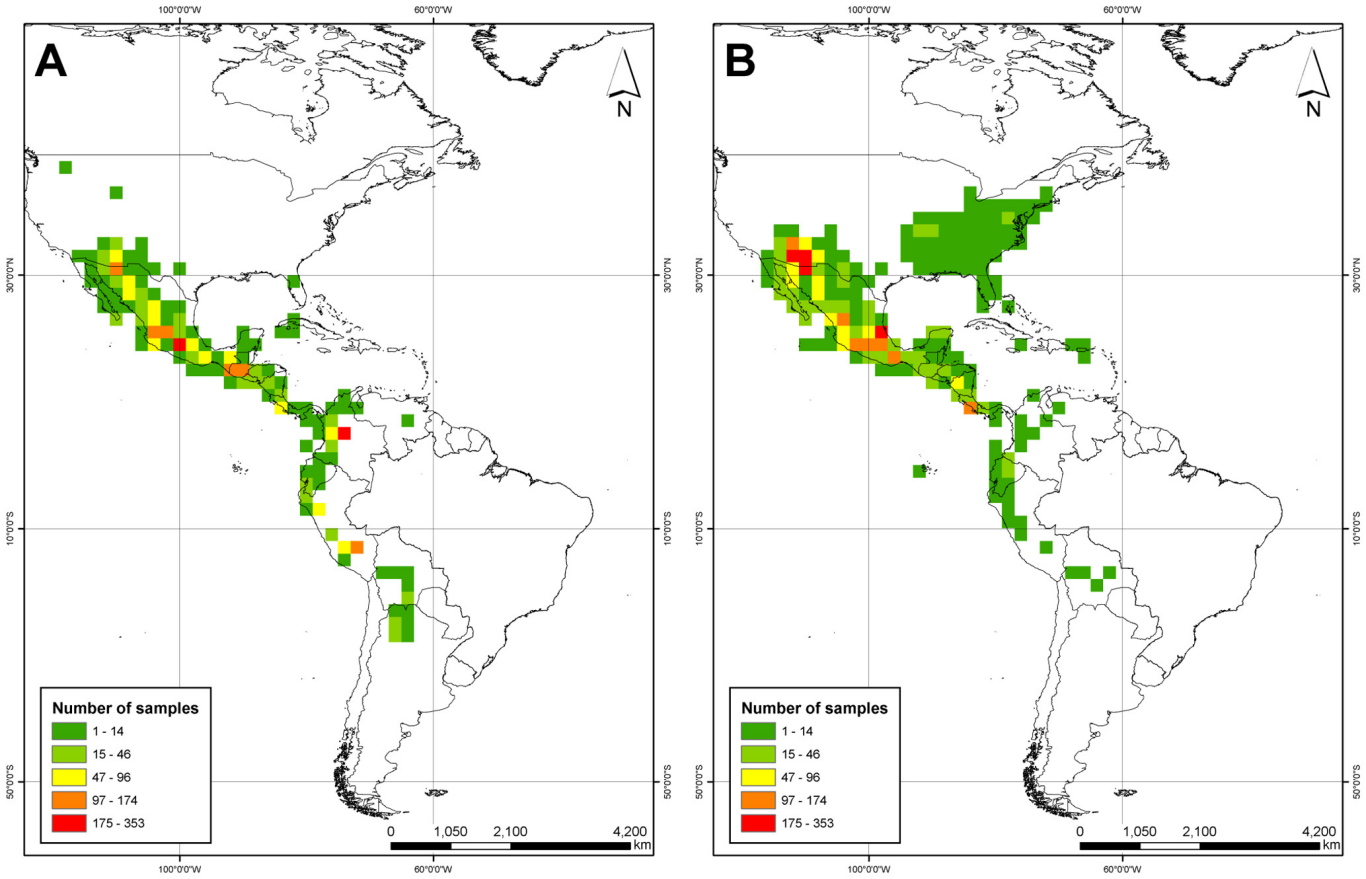
Density of sampling (sampling richness) for (A) genebank assessions and (B) herbarium records for *Phaseolus*.

Germplasm collections of the Phaseolus genepool are not distributed equally in relation to total herbarium collections (Figure 1). The number of genebank accessions in a 200 km cell ranged from 1 to 273, while that of herbarium collections ranged from 1 to 373. Observable differences in the two maps (gaps) are present in the eastern United States, Costa Rica, Nicaragua, and in the north of Mexico and along its border with United States. Most of the areas in central Mexico are however well sampled and it is possible that species occurring in those areas are adequately conserved. This was also observed in some areas in South America (particularly in the Colombian, Ecuadorian and Peruvian Andes), where a greater proportion of genebank accessions have been collected, potentially indicating a better coverage of taxa in genebanks for populations from these regions.

### 2. Sampling deficiencies at the taxa level

Of 85 taxa, 35 (41.2%) had no germplasm accessions, 26 taxa (30.6%) had 1–9 accessions, and 24 taxa (28.2%) had 10 or more accessions. From the 85 taxa, 61 (71.8%) taxa presented a SRS below 3, indicating poor representativeness of the number of genebank accessions in relation to herbarium collections, whilst 16 taxa (18.8%) showed SRS between 3.01 and 5, 4 (4.7%) between 5.01 and 7.5, and 4 (4.7%) greater than 7.5.

**Figure 2:**
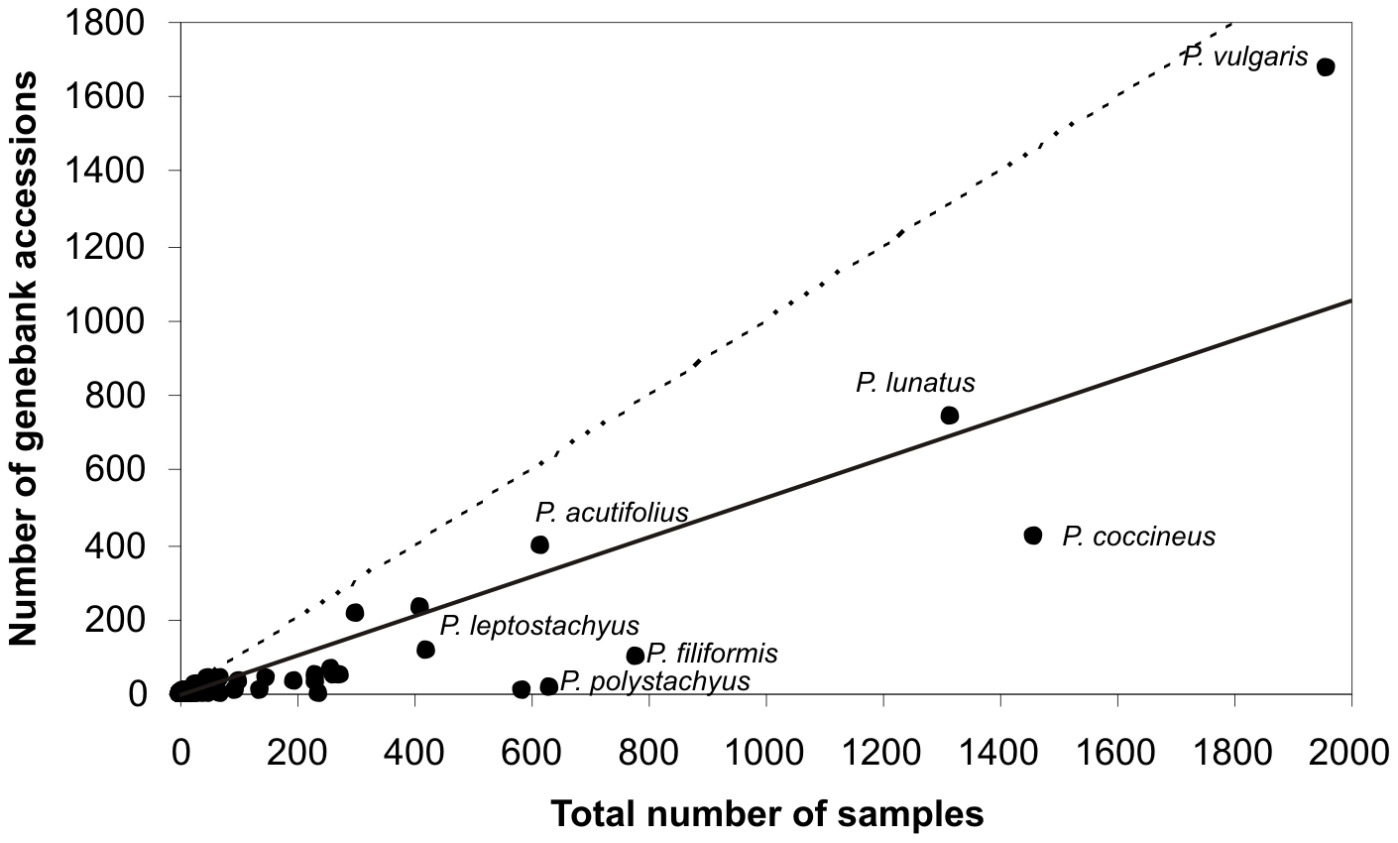
Number of genebank accessions versus all samples (genebank accessions plus herbarium specimen records).

The total representativeness (only in terms of the total number of samples, Figure 2 –intermittent line) is above the average representativeness of germplasm collections (continuous line), signifying that on average, species are likely to have fewer genebank accessions than herbarium specimens. *P. vulgaris*, *P. acutifolius* and *P. lunatus* appear well conserved in relation to both the gross number of accessions (compared to other taxa), and in proportion to their respective number of herbarium records.

### 3. Potential distribution models for taxa

We used high-resolution global climatic datasets developed by Hijmans et al. [72]. WorldClim includes monthly data at 30 arc-seconds resolution (approximately 1 km near the Equator) for total precipitation, and mean, maximum and minimum temperatures. Using such monthly datasets, 19 bioclimatic variables have been derived [73], representing average yearly climates, stressful and extreme conditions, and interannual seasonality (Table 2).

**Table 2:** List of derived bioclimatic variables used in the analysis.

ID	Variable name	Units
P1	Annual mean temperature	°C
P2	Mean diurnal temperature range	°C
P3	Isothermality	N/A
P4	Temperature seasonality (standard deviation)	°C
P5	Maximum temperature of warmest month	°C
P6	Minimum temperature of coldest month	°C
P7	Temperature annual range	°C
P8	Mean temperature of wettest quarter	°C
P9	Mean temperature of driest quarter	°C
P10	Mean temperature of warmest quarter	°C
P11	Mean temperature of coldest quarter	°C
P12	Annual precipitation	mm
P13	Precipitation of wettest month	mm
P14	Precipitation of driest month	mm
P15	Precipitation seasonality (coefficient of variation)	%
P16	Precipitation of wettest quarter	mm
P17	Precipitation of driest quarter	mm
P18	Precipitation of warmest quarter	mm
P19	Precipitation of coldest quarter	mm

We downloaded WorldClim data at 30 arc-seconds, calculated the bioclimatic indices and aggregated the 30 arc-seconds datasets to 2.5 arc-minutes using a bilinear interpolation in order to reduce the computational time and data storage needs. Although most of the bioclimatic indices used to develop the niche models are highly correlated (particularly in the tropics), we used the complete set of 19 bioclimatic variables in Table 2 because (1) they are useful to provide the best possible description of the climatic requirements of species during a single average year, (2) these correlations might not hold in space and time, (3) the alternative approach of dropping some variables leads to underestimation of distributions and poor performance of Maxent [62], (4) the alternative approach of reducing the set of variables to a subset of orthogonal vectors [60] might lead to loss of valuable climatic information and tends to complicate the interpretation of results of the application of the niche model, and (5) the Maxent model prevents over-fitting due to the use of a set of correlated environmental predictors by assigning weights based on the relative importance of the variable to the model [55], [59], [61].

The geographic distributions of 51 out of the 85 taxa were considered sufficiently accurate and stable to be mapped. Potential distribution coverage was estimated via the convex hull method for 3 additional taxa (*P. marechalii*, *P. salicifolius*, and *P. rotundatus*). Therefore, a total of 54 taxa were assessed further.

**Figure 3:**
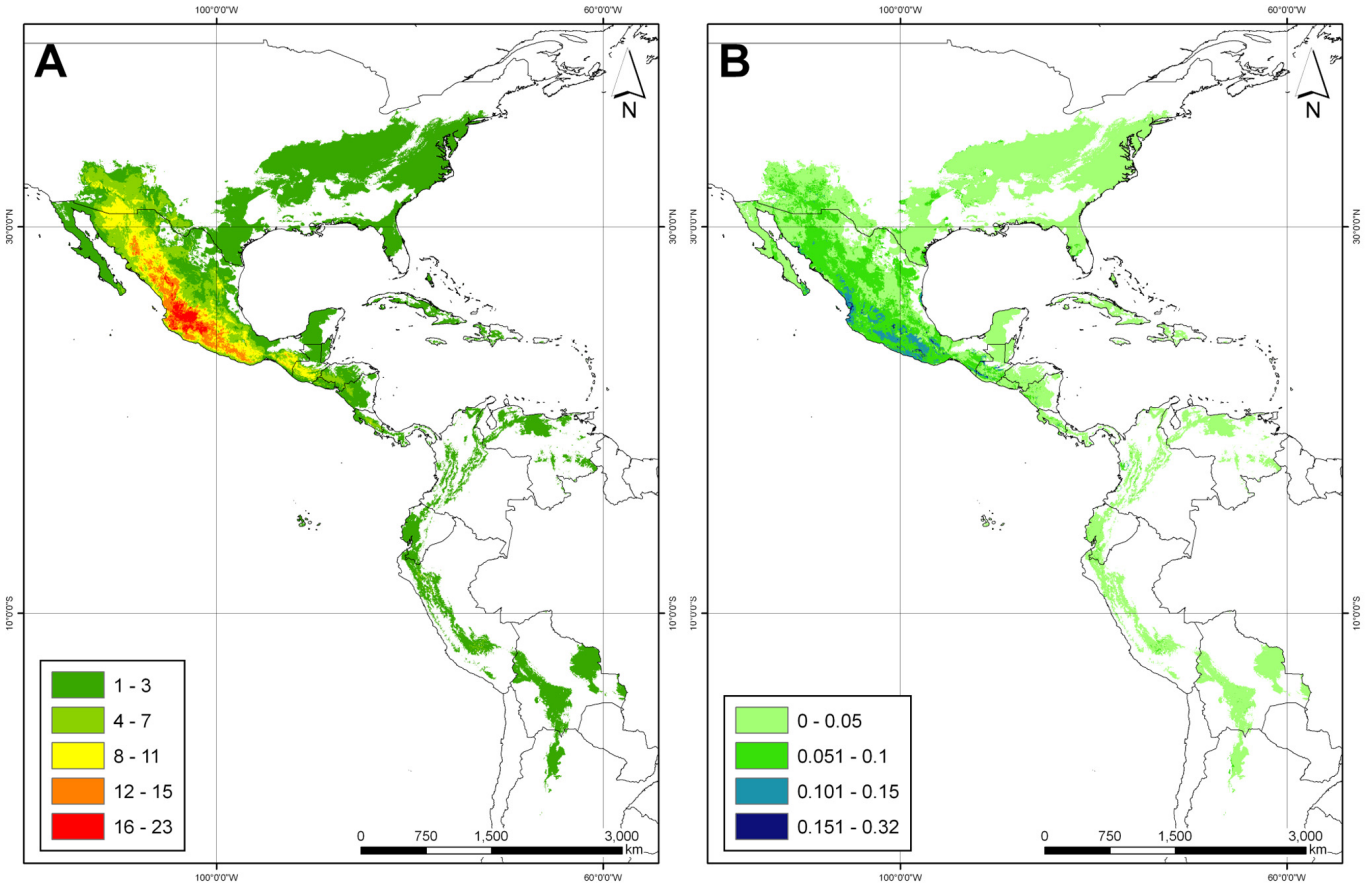
Species distributions and uncertainties. (A) Taxon richness for *Phaseolus* based upon potential area of distribution of all taxa, (B) modeling uncertainties as maximum standard deviations among all modeled taxa.

The genus was modeled to occur from the northern border of the United States through Central America, and along the Andean chain into northern Argentina (Figure 3a). Potential taxon richness ranged from 1 to 23 taxa per grid cell. Taxon diversity hotspots were mainly found in southern and western Mexico and in the southern United States, as well as some highland areas of Guatemala, Honduras and Costa Rica, where 6 to 11 taxa are potentially distributed in a single 5 km pixel.

Uncertainties in modeling distributional range calculated by the maximum standard deviation among any possible class (i.e. taxon) varied from 0 to 0.32 (Figure 3b), with the vast majority of the potential distribution coverage of the genus presenting a modeling uncertainty below 10%, and only very few areas presenting more than 15% variation in predicted probabilities. High uncertainty areas do not coincide with high diversity areas, confirming the reliability of the Maxent algorithm in predicting the geographic distributions of our set of taxa. These small spots are located in southwestern Mexico along the very western edge of Nayarit (municipalities of El Nayar, Rosamorada, Tepic), along the borders of Guerrero and Oaxaca, in northern Oaxaca, and in northeastern Michoacán. Despite the observed uncertainties, these areas with more than 15% variability among predictions account to less than 10% of the total potential distribution coverage of the genus.

### 4. Geographic coverage assessment

**Figure 4:**
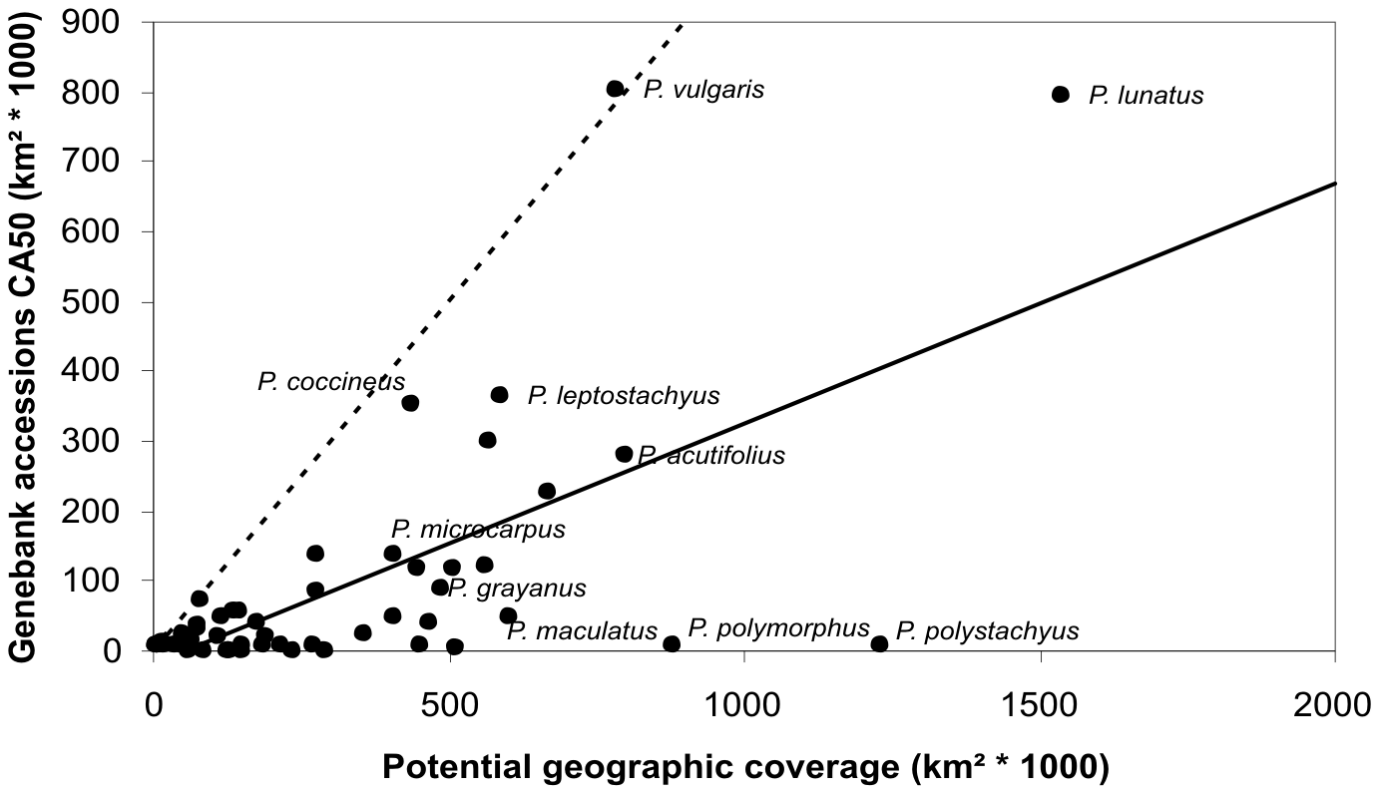
Geographic coverage of genebank accessions against total potential distribution coverage of taxa.

The comparison between the CA50 and the size of the potential distribution showed that there are 30 taxa out of the 54 assessed (55.6%) with GRS below 3.01 (less than 30% of representativity in terms of geographic coverage), 12 taxa (22.2%) with GRS between 3.01 and 5, 4 taxa (7.1%) with GRS between 5.01 and 7.5, and 8 taxa (14.8%) with GRS greater than 7.5. The great majority of taxa have germplasm collections covering a geographic range considerably smaller than the potential geographic area in which the taxon is distributed (Figure 4), thus indicating the need for further collecting in order to fill geographic gaps.

The average representativeness line (intermittent line) is above the complete representativeness line (continuous line), indicating that the representativeness of germplasm collections in comparison to the total potential distribution coverage is low on average, and relatively high only for a few species (namely the wild progenitors *P. vulgaris*, *P. coccineus*, *P. acutifolius* and *P. lunatus*).

### 5. Determination of environmental gaps

The principal components analysis showed that the first two components explained up to 81.5% of the total spatial variability among the *Phaseolus* genepool target area (61.2 and 20.3% for PC1 and PC2 respectively). Re-scaling of these two components' weights resulted in a weight of 75.03% for PC1 and 24.97% for PC2. Out of the 54 modeled taxa, 10 (18.5%) presented ERS below 3.01, indicating a significantly low environmental representativeness (i.e. less than 30%) in germplasm collections; 7 (13%) taxa presented an ERS between 3.01 and 5; 7 taxa (13%) between 5.01 and 7.5; and 30 taxa (55.6%) above 7.5. Notably, environmental representativeness of genebank accessions was found to predominantly fit in the two extreme classes (below 30% and above 75%) for most of the taxa.

**Figure 5:**
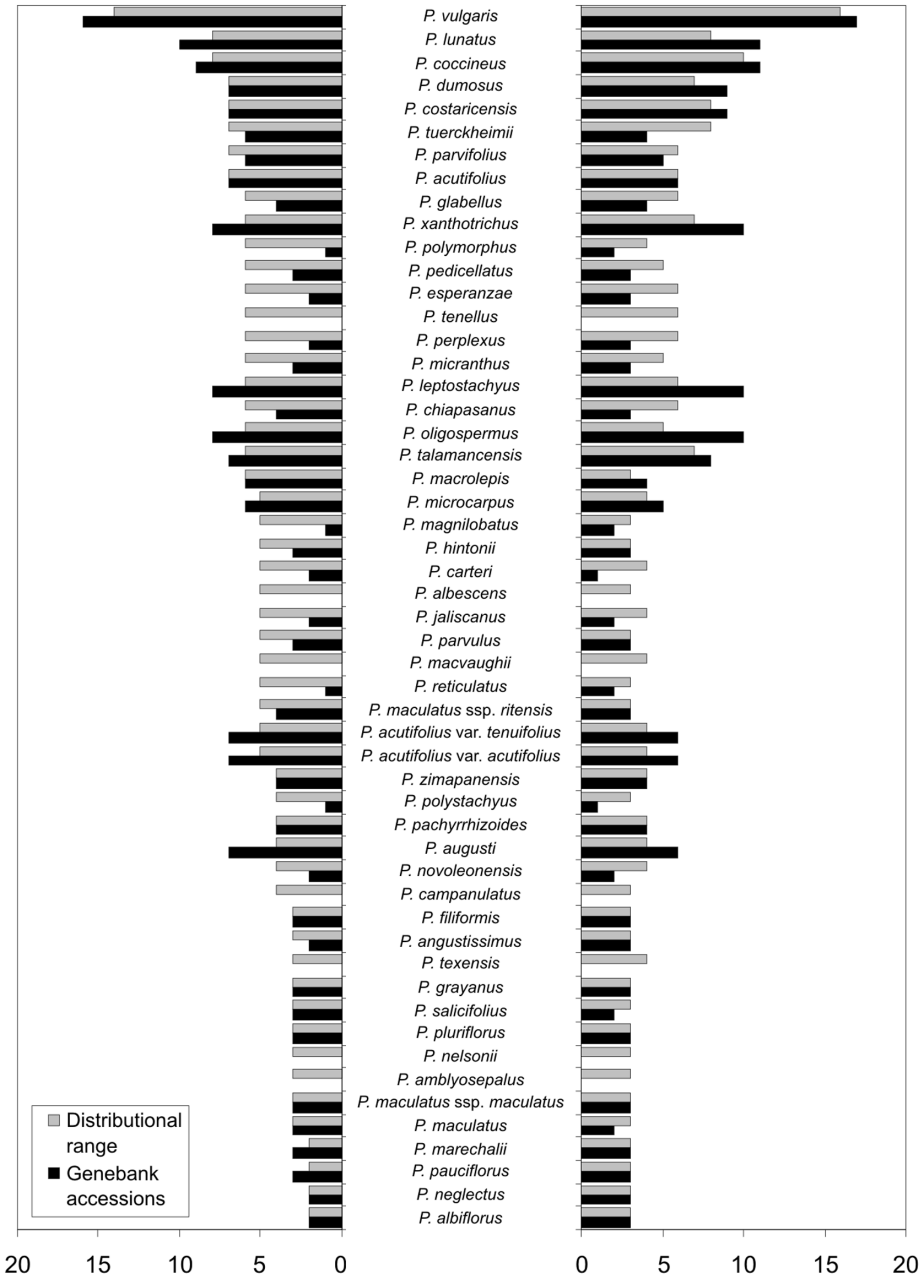
Coverage of genebank accessions versus potential environmental area for modeled species for the first (left) and second (right) principal components.

*P. vulgaris* and *P. lunatus* showed the highest coverage of potential environmental range, with 8 and 14 respectively out of the 20 classes along PC1, and 8 and 16 classes along PC2 (Figure 5). Germplasm representativeness of these environmental classes is for both species significantly high (90% or more representativeness in both classes). For wild *P. vulgaris*, among other cases (Figure 5), we found the environmental distribution of genebank accessions to be broader than the environmental distribution of the potential distribution coverage, which may be explained as an artifact given the use of the ROC-plot based threshold for binning the species distributions (i.e. the omission rate), the native area (i.e. one or two small localities where the taxon occurs might not be reported in literature), or the use of the CA50 around germplasm locations, which might enlarge the range towards unsuitable habitats, particularly where the landscape changes rapidly (e.g. topographically diverse regions, such as the Andes). A broad range of adaptation to climatic conditions may be covered by current germplasm collections, but it should be noted that small environmental gaps remain even for these well-sampled species.

### 6. Rarity of each species

Rarity of species was not included in the analysis since there were significant sampling biases that would lead to inaccurate results. In order to produce accurate results, the weight of the ERTS was finally established at 0.05, which is practically irrelevant and thus the step was dropped. If a subset of species with reliable sampling were to be analyzed separately (e.g. the five wild progenitors of the domesticated species), however, the ERTS could be calculated and weighted equally with the other scores when calculating the FPS.

### 7. Numeric assessment to determine the priority of collecting for *ex situ* conservation for each taxon

Out of the 85 taxa under analysis, 48 (56.5%) are either under-represented or not represented in any way in genebanks and therefore flagged as HPS for collecting (Table 3, Table S1). Of these taxa, 35 had no germplasm accessions, and 11 are listed as HPS due to the average of gross representativeness, geographic, and environmental gaps (FPS below 3.01). A further 2 taxa (*P. sinuatus* and *P. altimontanus*) couldn't be assessed due to uncertainties in the modeling and the data, and are included as HPS due to the need for collecting in order to provide adequate data for a gap analysis.

**Table 3:** List of taxa and available data included in the analysis (see Table S1 for full details).

Taxon	HS^1^	HS (RP)^3^	GA^2^	GA (RP)^3^	Total	Total (RP)^3^	FPS (GAP)	Class (GAP)^4^	EPS (DGD)	Class (DGD)^4^
**Sect**. *Acutifolii*										
*P. acutifolius*	219	119	396	67	615	186	5.7	LPS	NA	NA
*P. acutifolius* var. *acutifolius*	87	75	211	81	298	154	6.8	LPS	4	MPS
*P. acutifolius* var. *tenuifolius*	177	103	232	93	409	188	6.1	LPS	5	MPS
*P. parvifolius*	62	56	37	22	99	74	4.5	MPS	4	MPS
**Sect**. *Bracteati*										
*P. macrolepis*	24	6	3	3	27	6	8.3	NFCR	4	MPS
*P. talamancensis*	13	4	2	1	15	4	7.5	LPS	6	LPS
**Sect**. *Brevilegumeni*										
*P. campanulatus*	4	4	0	0	4	4	0.0	HPS	0	HPS
*P. oligospermus*	26	22	13	11	39	33	5.8	LPS	3	HPS
*P. tuerckheimii*	43	24	3	2	46	26	3.5	MPS	3	HPS
**Sect**. *Chiapasana*										
*P. chiapasanus*	53	8	3	3	56	8	4.1	MPS	2	HPS
**Sect**. *Coccinei*										
*P. coccineus*	1041	356	417	206	1458	560	7.3	LPS	4	MPS
**Sect**. *Coriacei*										
*P. maculatus*	106	62	39	17	145	79	4.0	MPS	NA	NA
*P. maculatus* ssp. *maculatus*	203	138	30	18	233	151	4.5	MPS	4	MPS
*P. maculatus* ssp. *ritensis*	190	120	68	30	258	150	4.6	MPS	2	HPS
*P. novoleonensis*	4	3	2	1	6	3	3.6	MPS	2	HPS
*P. reticulatus*	6	4	2	2	8	6	2.3	HPS	3	HPS
P. venosus++	10	6	0	0	10	6	0.0	HPS	0	HPS
**Sect**. *Digitati*										
*P. albiflorus*	49	4	1	1	50	4	4.3	MPS	6	LPS
P. albiviolaceus+	1	1	0	0	1	1	0.0	HPS	2	HPS
P. altimontanus#	2	2	2	2	4	2	NA	HPS	4	MPS
*P. neglectus*	9	6	0	0	15	11	0.0	HPS	2	HPS
P. trifidus++	1	1	0	0	1	1	0.0	HPS	NA	NA
**Sect**. *Falcati*										
*P. leptostachyus*	308	170	115	102	423	270	6.7	LPS	4	MPS
*P. macvaughii*	11	7	1	1	11	7	1.4	HPS	2	HPS
*P. micranthus*	21	9	2	1	23	10	2.1	HPS	4	MPS
P. opacus++	4	1	0	0	4	1	0.0	HPS	NA	NA
P. persistentus+	1	1	0	0	1	1	0.0	HPS	0	HPS
**Sect**. *Minkelersia*										
P. amabilis++	8	1	0	0	8	1	0.0	HPS	0	HPS
*P. amblyosepalus*	10	10	0	0	10	10	0.0	HPS	0	HPS
P. anisophyllus++	2	2	0	0	2	2	0.0	HPS	0	HPS
*P. nelsonii*	38	32	0	0	38	32	0.0	HPS	2	HPS
*P. parvulus*	168	101	29	17	197	118	3.2	MPS	2	HPS
*P. pauciflorus*	234	161	4	2	238	163	4.4	MPS	2	HPS
*P. perplexus*	11	7	2	1	13	8	1.7	HPS	3	HPS
P. plagiocylix++	4	2	0	0	4	2	0.0	HPS	0	HPS
*P. pluriflorus*	86	56	10	7	96	63	4.0	MPS	3	HPS
*P. tenellus*	21	9	2	1	22	9	1.0	HPS	0	HPS
**Sect**. *Paniculati*										
P. acinaciformis+	1	1	0	0	1	1	0.0	HPS	0	HPS
P. albinervus++	3	1	0	0	3	1	0.0	HPS	0	HPS
*P. augusti*	27	15	43	39	70	54	7.4	LPS	7	LPS
*P. jaliscanus*	66	12	2	1	68	12	1.8	HPS	2	HPS
P. juquilensis+	1	1	0	0	1	1	0.0	HPS	0	HPS
P. lignosus+	2	2	0	0	2	2	0.0	HPS	0	HPS
P. longiplacentifer+	1	1	0	0	1	1	0.0	HPS	0	HPS
*P. lunatus*	575	275	742	342	1317	616	6.9	LPS	4	MPS
P. maculatifolius++	2	1	0	0	2	1	0.0	HPS	0	HPS
*P. marechalii*	10	4	5	2	15	4	8.3	NFCR	3	HPS
P. mollis++	14	6	0	0	14	6	0.0	HPS	0	HPS
P. nodosus++	2	2	0	0	2	2	0.0	HPS	2	HPS
*P. pachyrrhizoides*	5	2	21	20	26	22	7.8	NFCR	8	NFCR
*P. polystachyus*	580	344	6	2	586	346	0.9	HPS	2	HPS
P. rotundatus++	3	2	1	1	3	2	6.7	LPS	5	MPS
*P. salicifolius*	10	3	1	1	11	4	7.2	LPS	0	HPS
P. scrobiculatifolius+	1	1	0	0	1	1	0.0	HPS	0	HPS
P. sinuatus#	76	12	1	1	77	12	NA	HPS	2	HPS
P. smilacifolius++	13	2	0	0	2	1	0.0	HPS	0	HPS
P. sonorensis++	16	3	0	0	16	3	0.0	HPS	0	HPS
P. xolocotzii++	1	1	0	0	1	1	0.0	HPS	0	HPS
**Sect**. *Pedicellati*										
P. dasycarpus++	5	5	0	0	5	5	0.0	HPS	0	HPS
*P. esperanzae*	26	15	7	7	33	15	4.4	MPS	2	HPS
*P. grayanus*	184	77	49	36	233	113	5.0	MPS	3	HPS
P. laxiflorus++	5	1	0	0	5	1	0.0	HPS	0	HPS
P. oaxacanus++	6	2	0	0	6	2	0.0	HPS	3	HPS
*P. pedicellatus*	129	71	8	8	137	79	2.9	HPS	4	MPS
*P. polymorphus*	23	5	1	1	24	6	1.4	HPS	3	HPS
P. purpusii++	5	1	0	0	5	1	0.0	HPS	0	HPS
P. scabrellus+	4	4	0	0	4	4	0.0	HPS	2	HPS
P. teulensis+	1	1	0	0	1	1	0.0	HPS	0	HPS
*P. texensis*	7	6	0	0	7	6	0.0	HPS	3	HPS
**Sect**. *Phaseoli*										
*P. albescens*	8	8	0	0	8	8	0.0	HPS	2	HPS
*P. costaricensis*	64	44	4	3	68	46	6.6	LPS	6	LPS
*P. dumosus*	52	14	9	7	61	14	6.5	LPS	5	MPS
*P. vulgaris*	284	209	1674	452	1958	661	8.9	NFCR	7	LPS
**Sect**. *Revoluti*										
P. leptophyllus+	6	1	0	0	6	1	0.0	HPS	0	HPS
Sect. *Rugosi*										
*P. angustissimus*	617	269	17	8	634	275	2.8	HPS	2	HPS
*P. carteri*	8	3	5	2	13	4	3.9	MPS	2	HPS
*P. filiformis*	682	397	98	46	780	441	4.6	MPS	2	HPS
**Sect**. *Xanthotricha*										
P. esquincensis++	4	3	0	0	4	3	0.0	HPS	0	HPS
P. gladiolatus++	1	1	0	0	1	1	0.0	HPS	3	HPS
*P. hintonii*	12	7	11	7	23	14	4.3	MPS	2	HPS
*P. magnilobatus*	16	7	2	1	18	8	1.6	HPS	2	HPS
*P. xanthotrichus*	11	8	38	30	49	38	9.0	NFCR	5	MPS
*P. zimapanensis*	10	5	16	13	26	17	7.3	LPS	6	LPS
**Not classified**										
*P. glabellus*	128	42	15	10	160	42	3.7	MPS	5	MPS
*P. microcarpus*	223	161	51	35	274	193	5.1	LPS	4	MPS

1 Number of herbarium specimens,

2 Number of genebank accessions,

3 Refers to the number of populations (unique locations identified) represented by the set of samples,

4 Prioritization of taxa is done as follows: HPS: High priority species, MPS: Medium priority species, LPS: Low priority species, NFCR: No further urgent conservation required. FPS indicates the result of the method proposed in this paper, and EPS indicates the prioritization given by expert knowledge (based on Daniel G. Debouck's expertise in *Phaseolus*).

+Indicates that the taxon had no genebank accessions and no herbarium samples with coordinates or location data;

++indicates a taxon for which a Maxent model was not possible and for which 0-few genebank accessions were available;

#indicates a taxon with some genebank accessions but no or limited herbarium samples with coordinates or location data. These taxa are listed as HPS for further collecting in order to inform the gap analysis.

Medium priority for further collecting was given to 17 taxa (20%), 15 taxa (17.7%) were given low priority, and only 5 taxa (*P. macrolepis*, *P. marechalii*, *P. pachyrrhizoides*, *P. xanthotrichus* and *P. vulgaris*) were assessed as well represented in *ex situ* collections.

### 8. Prioritization of geographic areas for collecting germplasm

**Figure 6:**
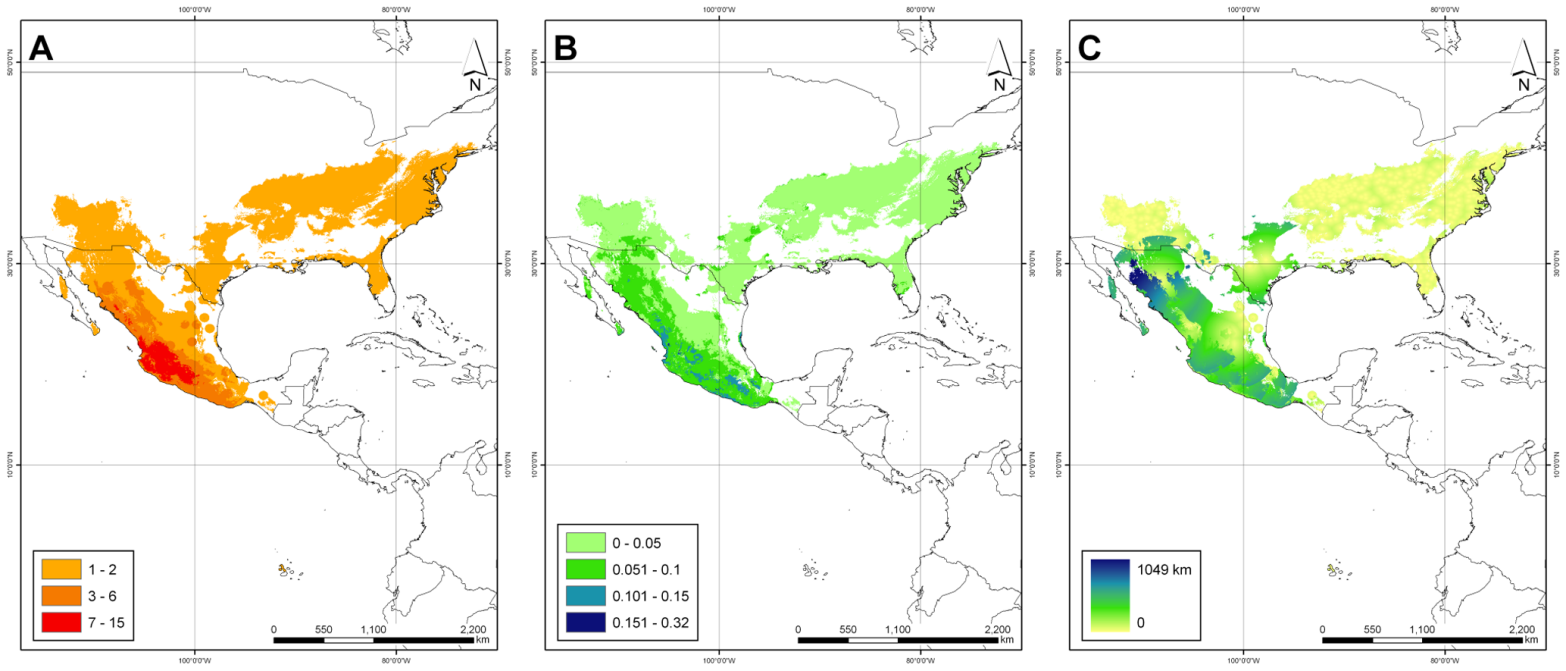
Prioritization results. (A) Zones where gaps in *ex situ* collections for multiple taxa overlap (collecting gap richness) for high priority species, (B) modeling uncertainties as standard deviations among high priority modeled taxa, (C) collecting uncertainties as maximum geographic distance to nearest known population.

36 priority taxa (i.e. those flagged as high priority and with sufficient location data) were mapped together, along with standard deviations on predicted Maxent probabilities (aggregated for all the taxa using the maximum value) and distances to the nearest population (also aggregated) (Figure 6). Potential collection sites have a richness of up to 7 taxa per grid (Figure 6a). Zones where gaps in *ex situ* collections for many *Phaseolus* taxa overlap are concentrated in central-western Mexico, with an extension along the Sierra Madre Occidental north to Sonora.

Andean environments where *Phaseolus* species are likely distributed appear in general to be adequately represented in genebanks for most of the species. Note that the narrow endemic nature of many of the under- or un-sampled taxa results in a need for very finely targeted collection trips to specific regions outside of the gap richness areas identified, for example to collect from populations of *P. carteri, P. novoleonensis*, and *P. plagiocylix* in isolated regions of Mexico, and *P. mollis* in South America.

The maximum modeling uncertainty (given by the maximum standard deviation of the 25 folds per taxon) was slightly greater than 15% in a very small area (dark blue spot in western Nayarit, Figure 6b). Interestingly, modeling uncertainties of high priority taxa had a maximum of 19%, significantly lower than uncertainties of the whole set of taxa under analysis (Figure 3b), strengthening confidence in results regarding high priority taxa. The distance to verified populations (Figure 6c) was greatest (i.e. uncertainty) in northwestern Mexico (southern Sonora, northern Sinaloa, and southwestern Chihuahua). The areas identified in these uncertainty analyses are least likely to contain target species.

### Comparison with expert opinion

The expert authority for *Phaseolus* was Daniel G. Debouck (DGD), head of the Genetic Resources Unit at the International Center for Tropical Agriculture (CIAT), author and co-author of numerous publications on *Phaseolus*, including a survey of the *Phaseolus* genepool in North and Central America [30], who has participated in many collecting missions for the genus throughout the Americas and has extensive expertise in taxonomy (including research at 67 different herbaria in the last 32 years), ecogeographic distributions, and level of *in situ* and *ex situ* conservation of the genepool.

DGD did not assess 4 taxa: *P. maculatus*, and *P. acutifolius* since he considered it enough to assess the subspecies and/or variants, and *P. trifidus* and *P. opacus*, since he considered them as doubtful taxa. All figures below are thus based on the total number of taxa assessed by DGD (81). Further taxonomic analyses of these species are needed in order to inform conservation priorities.

**Figure 7:**
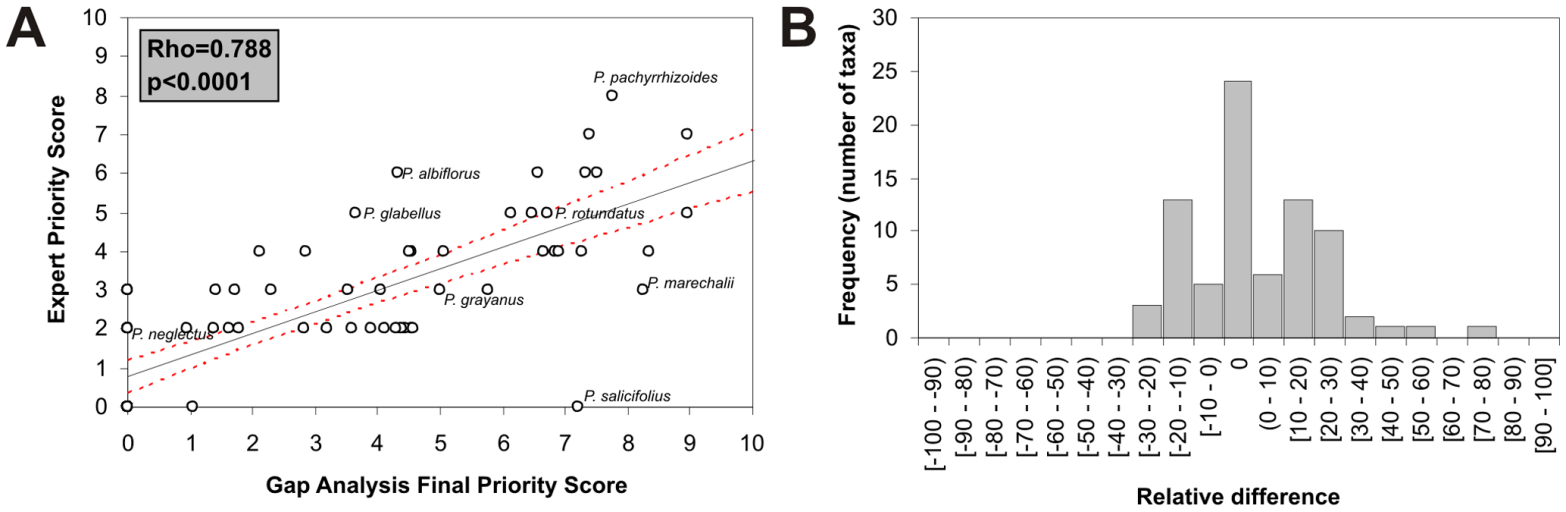
Validation results. (A) Frequency distribution of the relative difference [RD] and (B) linear trend between Final Priority Score (FPS) and Expert Priority Score (EPS) (the red dotted line indicates a 95% confidence interval).

In comparison to expert opinion, the gap analysis approach tended to underestimate priority for collecting in a considerable number of cases (30.9% of the taxa); however, scores for 28 taxa (34.6%) did align with expert opinion (with 0 as score for 24 of these). For 51 taxa (63%), the method and DGD agreed on the priority class, and from the remaining proportion, the difference was of one single class. In addition, the relative difference (RD) varied from −50% to 72.2% and the maximum difference between our approach and the expert's concept was around 7 units in the priority scale of 10 units. Moreover, 87.7% of the validated taxa (81) presented differences lower than 30% or greater than −30%, and only 2 taxa presented more than a 50% or less than −50% difference (*P. salicifolius* with 7.2 in EPS and 0 in FPS, *P. marechalii* with 8.3 in EPS and 3 in FPS). Only *P. salicifolius* was found to have more than 70% difference between EPS and FPS (Figure 7a).

The linear trend between EPS and FPS has a Spearman correlation coefficient of 0.79 (p<0.0001, n = 79). However, as previously stated, the gap analysis approach tends to underestimate the priorities compared to expert opinion (average underestimation is −10.7%, Figure 7b).

A number of taxa fall far from the linear trend (i.e. *P. neglectus*, *P. albiflorus*, *P. salicifolius* and *P. pachyrrhizoides*). Whilst for *P. pachyrrhizoides* this is due to a very high accuracy (ERS and FPS are equal) in comparison with the propagating error in the regression line (i.e. the underestimation error), differences for other taxa generally result from lack of geographic data for a robust gap analysis, likely taxonomic misidentifications in records, and/or difficulty in eliminating duplicates in records (e.g. *P. neglectus*, *P. albiflorus*).

For species such as *P. xanthotrichus* and *P. oligospermus*, the gap analysis approach indicated little need for further collection, as germplasm has been collected throughout the most of the region of recorded herbarium collections and environments occupied by those collections. However, expert knowledge on other areas of distribution of the species, under-recorded in online herbarium data, gave the species higher priority on the EPS.

## Discussion

Success of the gap analysis method in identifying priority taxonomic, geographic, and environmental gaps is directly dependent on the quality of input data and robustness of the modeling based upon the data. In this section we discuss uncertainties and limitations concerning the method:

### a. Input data availability, bias and certainty

The quality of the input geographic information (i.e. climatic and occurrence data) directly affects the performance of species distributions models [53], [57], [58], [60], [62], [74]. Geographic data for specimens is generally less than optimal and is unevenly distributed across taxa, due to the bias of collecting activities toward particular species or locations, a historically insufficient prioritization of recording and maintaining of geographic data, lack of high quality absence data for species, and limited accessibility of stored data for some collections. Many regions of the world remain un- or under-sampled, particularly highly inaccessible areas, and those chronically affected by war or civil strife.

Recently described and/or under-studied taxa, such as P. acinaciformis, P. juquilensis, P. longiplacentifer, P. persistentus, P. scrobiculatifolius, P. teulensis, P. albiviolaceus, P. leptophyllus, P. lignosus, P. scabrellus, and P. sinuatus, may require further taxonomic clarification, and are generally in need of further collecting, and characterization of the collected populations, in order to clarify identification and facilitate accurate prioritization.

Infraspecific taxa (variants and subspecies), such as those of *P. maculatus* and *P. acutifolius*, may also be incompletely treated in the analysis due to data constraints. There are several records of these species that remain undetermined at the infraspecific level. Due to overlapping ranges of distribution for various infraspecific taxa, unassigned records cannot be easily differentiated based on collection location. In the gap analysis we have therefore assessed both the species level and the infraspecific taxa.

More germplasm of *Phaseolus* may be conserved worldwide than the accession data used in this analysis indicate, as the data from some genebanks was not accessible. We assume that, with few exceptions, the accessions whose data was not accessible are also generally inaccessible to crop breeders and researchers worldwide. Areas where these collections were made may not represent a gap for the particular holding collection, yet they are effectively a very real gap for rest of the global community.

Duplication between and within institutes might inflate the numbers of unique records for some of the taxa, leading to bias in the prioritization results. The use of different numbering systems, and lack of tracking of former records, leads to an overestimation of samples held, and difficulty in identifying duplicates, perhaps especially for the most commonly exchanged species (e.g. wild progenitors). For *Phaseolus*, we found that large differences can exist between the number of records and of actual populations both for genebanks (up to 83.1%) and herbaria (up to 87.5%). The data preparation phase of the analysis involved a thorough identification of duplicates in order to avoid inflation of numbers of records and therefore biases in prioritization. Further, the geographic representativeness score (GRS) takes distinctness/uniqueness of populations into account indirectly, and the environmental representativeness score (ERS) addresses the issue by illuminating gaps in the abiotic adaptations of the sampled material (i.e. number of different climatic environments covered by the conserved material).

Location data constraints may also limit the taxa for which the method may be applied, as well as lead to an underestimation of taxon distributions. From the 45 different data sources, 24 (53.3%) had more records without location data than with location data, and only 9 (20%) of the sources presented all of their records with coordinates or with detailed location data (Table 1). For genebanks, 71.1% of the data presented reliable location data and 28.9% had either no location data or location data were unreliable, whilst for herbaria, 70.2% of the data presented coordinates and 29.8% did not present any useful location data.

Additional data, such as absence of the taxon, would certainly improve model-training by increasing the model's ability to discriminate between presence and absence areas. These data are unfortunately not available in conventional genetic resources databases [59], [75]. Future collecting should be planned with an eye to the improvement of gap analysis approaches and should thus consider a more systematic recording of absences, geo-referencing all records, and making widely accessible data from all available germplasm and herbarium samples. These actions will improve the performance of species distribution models and any conclusions drawn from them.

### b. Ability of the species distribution model used (i.e. Maxent) to adequately predict the potential and realized niche of taxa

The Maxent modeling technique was chosen for its ability to handle sample bias and spatial autocorrelation of species data [57], [58], [64] so as to provide high confidence species distributions models even given limited or biased location data. Maxent is an algorithm known to reliably predict the potential niches of species, and has been tested by several authors under a wide range of conditions and configurations (e.g. 55, 61, 57–58, 63–65, among others); although we note that some reports [68], [76]–[77] consider niche modeling techniques misleading and of limited use in certain contexts. As the robustness of Maxent is considered in the publications listed above, we do not provide a full analysis here.

We used the average test-data AUC, which showed that 52 species distribution models were reliable (i.e. accurate and stable, Table S1). Using the current configuration, the AUC statistic is not likely to be biased by the pseudo-absences range [63]. Discrimination between presence and absence sites was therefore considerably positive for most of the taxa (∼70%). Particularly good was the performance of taxon distribution models with more than 40 data points.

Moreover, the uncertainties associated with the application of a probabilistic model such as Maxent and depicted by the 25-fold cross-validated models for each of the taxa indicated that standard deviations among predictions ranged from 0 to 0.19. Collecting priorities are more uncertain in limited areas (e.g. along the western coast of Mexico), but are relatively robust across the vast majority of the distributional range of the genepool.

However, there was a set of taxa (those marked with + in Table 3) for which we were not able to develop species distributions models due to either lack of samples or to the distribution of those samples. These species could benefit from other approaches, such as Bayesian techniques [78], which are able to develop probability surfaces even from a single point. Here we did not include these additional approaches, given the uncertainties involved with these models. We rather use specimen data (i.e. herbarium sampling points) to depict areas where these species can be potentially collected.

### c. Geographic collecting priorities

To analyze the validity of geographic gap results, we have calculated the stability (standard deviation) of the Maxent models and have also provided the distance to the nearest population within the collection zone (Figure 6).

Additional analyses, including threat level, can be incorporated into the methodology in order to refine conservation priorities. Possible threats that could lead to genetic erosion in wild species populations include fires, grazing pressure, invasive species, deforestation, habitat modification and degradation, urbanization, and climate change, among others [5]. Accession-level genetic data may also serve as an input in order to identify gaps in genetic diversity. Additional environmental data, such as soil type, may further define potential distributions of species. These additional inputs are currently only rarely available at high detail over large geographic areas or for all taxa in a genepool, but this may improve with the ongoing development of GIS and decreasing costs of genotyping. Taxon-specific knowledge may also be used to refine or weight priorities, giving some species higher importance in the final result (e.g. focusing on specific traits of interest, adjusting to phytosanitary/noxious weed constraints, recognizing legal constraints to access, prioritizing in order to capitalize on appropriate seasonal collecting windows, etc.)

In our approach, we include all wild relatives of the crop without regard to relatedness to cultivated species, weighting them equally, with the assumption that a wide range of taxa are potentially useful to provide genes for crop improvement [11], recognizing the lack of data on relatedness. Information on relatedness and threat level can be added to the prioritization exercise by experts with specific interests or familiar with local conditions.

When this is done for *Phaseolus* the following gaps are highlighted. Collecting a few (1–5) populations is needed for 35 taxa that currently have no genebank samples conserved. Out of the five wild progenitors of the domesticated species, *P. vulgaris* and *P. dumosus* have been relatively well sampled, and only small gaps remain to be filled. Briefly, gaps for wild *P. vulgaris* are present in: Oaxaca, El Salvador, Panama, western Andes of Venezuela, northern central Bolivia, and San Luis in Argentina. For wild *P. dumosus*: eastern Chiapas and Alta Verapaz in Guatemala. For the remaining three progenitors, the gaps are substantially more important. For *P. acutifolius*: Sonora, Chihuahua, many spots in western Mexico and in Guerrero. For *P. coccineus*: Chihuahua down to Guatemala. For *P. lunatus*, gaps exist throughout the very large range (from the Revillagigedo Islands, Baja California Sur and Sinaloa to Puerto Rico, and down to Salta and Formosa in Argentina.

Regarding the secondary genepool of each of the five cultigens: for common bean, runner bean, and year bean, additional collecting is needed for *P. albescens*, *P. costaricensis*, and *P. persistentus* (if placement into Sect. *Phaseoli* is confirmed). For tepary, collecting is needed for *P. parvifolius* (all across its range from Chihuahua down into Guatemala). For Lima bean, concerted effort is required because few (if any) accessions are available for taxa within Section *Paniculati*, as well as *P. maculatus*, *P. novoleonensis*, *P. reticulatus*, *P. ritensis*, and *P. venosus* within Section *Coriacei*.

For the remaining *Phaseolus* species (not highly related to any cultigen given molecular evidence available today), a few accessions exist for taxa such as *P. chiapasanus*, *P. esperanzae*, *P. pluriflorus*, and *P. micranthus*. Remaining species are in need of further collecting in order to secure germplasm *ex situ*.

### d. Comparison with expert knowledge

The method performed well as compared with expert knowledge on the *Phaseolus* genepool, 81.2% of the taxa presenting differences between −30% and 30%, and only one taxon with a difference of more than 70% between EPS and FPS. We note that although the expert will often refine the analysis by adding further insight and by qualifying data, the gap analysis also holds the potential to highlight taxonomic, geographic, and environmental gaps previously unknown to the expert.

In order to provide a more robust test, multiple experts could be consulted. As GIS approaches continue to expand and improve, a more comprehensive validation procedure may be performed with a network of experts, facilitated through an online portal.

Expert intervention within the gap analysis method is especially critical during (1) thorough taxonomical review of the genepool, including variants and/or subspecies changes according to the latest studies, (2) the full evaluation and georeferencing of locality names in the dataset, and (3) the further refining and correction of priorities when a data availability issue is detected.

Expert taxonomic knowledge will of course also be vital in the actual field collecting, especially for understudied species (e.g. *P. albinervus*, *P. leptophyllus*, and *P. purpusii*). This has proven to be important in this genus, as numerous new species have been identified only during germplasm collecting missions (e.g. *P. altimontanus*, *P. costaricensis*, *P. novoleonensis*, *P. persistentus*, *P. rotundatus*, and *P. talamancensis*).

### Conclusion and final remarks

This study proposes a method for the rational prioritization of taxa within a genepool for collection for *ex situ* conservation, using *Phaseolus* as a model. The method builds upon the standard comparison of herbarium samples with genebank accessions via gap analysis [17], yet aims to address sampling biases by modeling species distributions with a robust algorithm, and refining these distributions using two different criteria. Furthermore, the method identifies priorities based not only on taxonomic and geographic gaps, but also environmental gaps. Priority locations for sampling of gaps result, as well as gap richness models contributing to the identification of collection locations for maximum efficiency. The results cover the four target outcomes of gap analysis identified by Nabhan [18]. Collecting for *ex situ* conservation should prioritize the resulting taxa, including those not or under-sampled *ex situ*, as well as geographic and environmental gaps in the distribution of taxa with some degree of germplasm currently conserved.

We found 48 high priority taxa (56.5%) (Table 3, Table S1), 35 (41.1% of total) of these not recorded as represented *ex situ* by even a single accession. Acknowledging that the results for a number of these species may potentially be affected by data availability constraints, in the most optimistic case, around half of the taxa in the genepool are highly under-represented in *ex situ* conservation. There is therefore a clear need for further collecting in order to cover the full range of taxonomic, geographic and environmental diversity.

The greatest priority regions for further collecting are located in northern Central America (i.e. Mexico and Guatemala), with a maximum potential sampling richness of 7 species per 5 km cell. However, there are a number of species that require individually targeted efforts in other areas (e.g. *P. mollis*, in the Galapagos Islands).

Additional criteria, such as threats to taxa, and degree of relatedness of taxa to cultivated species, may also be included in the analysis, when data is sufficiently available. In order to include a more complete picture of conservation, the method should ideally be coupled with *in situ* gap analysis results [e.g. 15], i.e. comparison of distributions with the extent of protected areas. In general, the high priority taxa identified in the analysis are likely to be those also most highly prioritized for *in situ* conservation, although this was not explored in the current analysis.

The method is applicable to any set of related taxa, given adequate geographic data and a thorough taxonomic and geographic referencing process. Genepools whose taxonomy has not received sufficient attention (e.g. *Oryza* in the Americas), or which have not been well sampled for herbarium specimens, will present particular challenges in producing reliable results. As each genepool is different, the analysis must be adapted according to data availability, and tested against expert knowledge, preferably repeatedly. Once the method has been applied to a number of crop genepools, the prioritization of taxa and “gap richness” mapping may be applied for these genepools together, potentially facilitating the identification of priority regions (“plant genetic resource gap megacenters”) for the efficient and effective collecting of CWR diversity on a global scale.

## Supporting Information

Table S1 Complete set of metrics used for the assessment of species distributions and *ex-situ* conservation status. Prioritization of taxa is done as follows: HPS: High priority species, MPS: Medium priority species, LPS: Low priority species, NFCR: No further urgent conservation required. FPS indicates the result of the method proposed in this paper, and EPS indicates the prioritization given by expert knowledge (based on Daniel G. Debouck's expertise in Phaseolus). +Indicates that the taxon had no genebank accessions and no herbarium samples with coordinates or location data; ++indicates a taxon for which a Maxent model was not possible and for which 0-few genebank accessions were available; #indicates a taxon with some genebank accessions but no or limited herbarium samples with coordinates or location data. These taxa are listed as HPS for further collecting in order to inform the gap analysis.
(0.03 MB XLS)
